# Deeply Embedded Corneal Foreign Bodies With Fungal Keratitis Secondary to Rambutan Fruit Fall

**DOI:** 10.7759/cureus.22413

**Published:** 2022-02-20

**Authors:** Abdul-Hadi Rosli, Muhammad Yusuf Abdurrahman, Khairidzan Mohd Kamal

**Affiliations:** 1 Ophthalmology, Kulliyyah of Medicine of International Islamic University Malaysia, Bandar Indera Mahkota, MYS

**Keywords:** vegetative material, fungal keratitis, corneal foreign bodies, ocular trauma, nephelium lappaceum l

## Abstract

Rambutan is widely found in remote areas in Southeast Asia. It may lead to serious ocular trauma if it accidentally hits the eyes. This case report describes a patient who presented with deeply embedded cornea foreign body and fungal keratitis following direct ocular trauma by rambutan fruit. This report identified the features of this fruit that had the potential to cause serious ocular trauma. Ocular protection equipment is essential to prevent injury during harvesting the fruit.

## Introduction

Rambutan (*Nephelium lappaceum L.*) is a famous tropical fruit in the Southeast Asia region. It is widely grown in Malaysia and Indonesia [1]. It is covered with a soft spine having spinterns and usually grows in a group of sprigs, connected by a wooden stalk [1-2]. Even though it is a small fruit, due to its physical characteristics, it may inflict ocular trauma when it accidentally falls directly on the eyes while harvesting from the trees. Besides that, the study has shown the presence of endophytic bacteria in the rambutan fruit including Corynebacterium, Bacillus, Staphylococcus, and other species [3]. The study also showed the presence of fungus colonies in the rambutan fruit [4]. This causes rambutan-related ocular trauma prone to cause infections such as keratitis and endophthalmitis. Ocular trauma with vegetative material like rambutan fruit also can lead to fungal infection such as fungal keratitis [5]. We report a case of deeply embedded cornea wooden-foreign bodies and fungal keratitis following ocular trauma by rambutan fruit.

## Case presentation

A 61-year-old gentleman with underlying hypertension was referred for right ocular trauma following being hit by a rambutan fruit at home. He was looking up while harvesting a sprig of rambutan fruits under the tree when suddenly, one of the fruit directly fell over his right eye. He did not use any ocular protection equipment during the trauma. Post-trauma, he developed right eye pain with foreign body sensation. It was associated with reduced vision and tearing over the right eye. During the examination, the patient was alert and conscious. Hemodynamically he was stable. The visual acuity of the right eye was 6/15 and 6/6 in the left eye using the Snellen chart. There was no evidence of injury over the periorbital region and eyelids. Examination of the right eye showed diffusely injected conjunctiva. However, no entry wound was seen.

**Figure 1:**
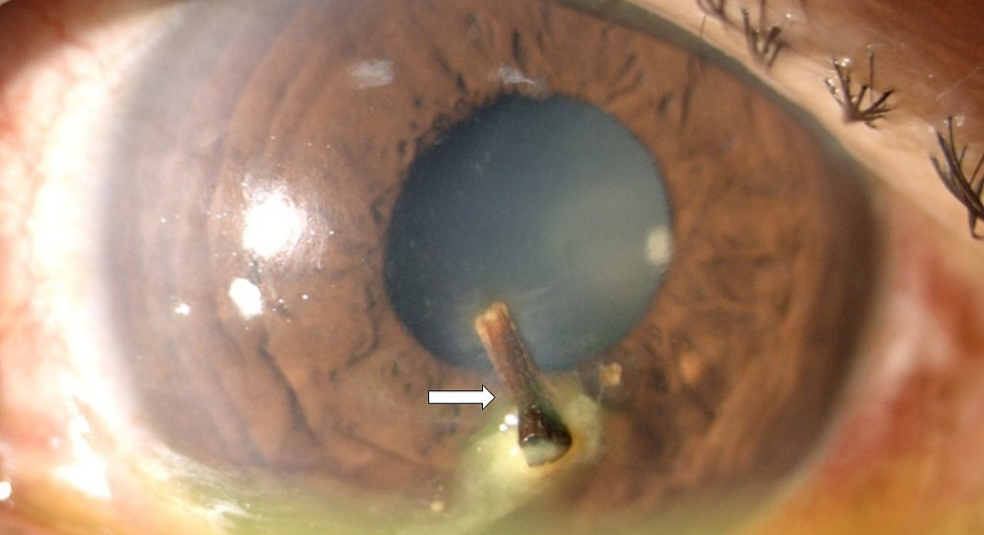
Deeply embedded cornea foreign body (arrow).

**Figure 2:**
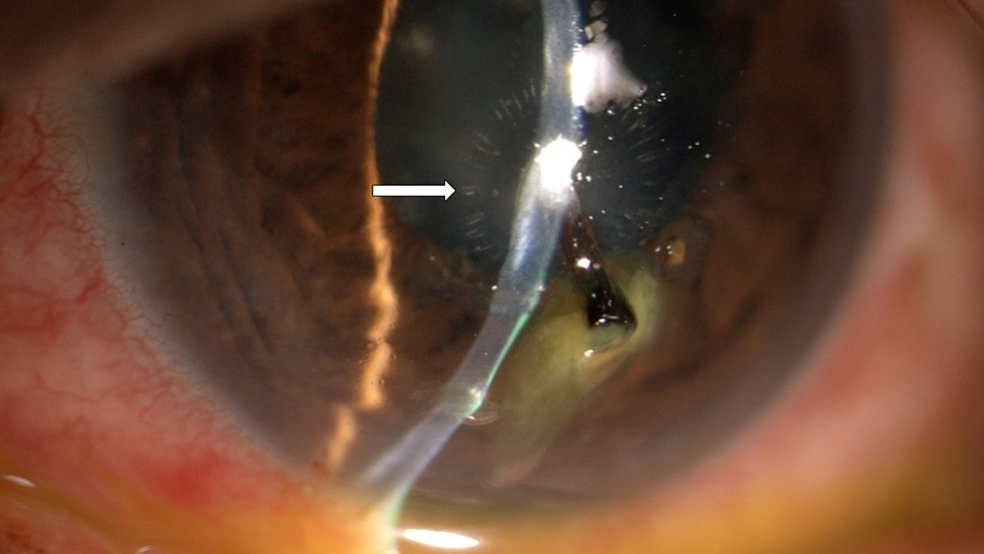
Foreign body impinge to the descemet membrane causing circumlinear striation (arrow).

There was a deeply embedded elongated woody-like cornea foreign body measuring about 0.5 mm length, at the paracentral region, 6 o'clock nearing involves the visual axis (Figure 1). It was deeply embedded up to the posterior stroma layer and impinging to the Descemet membrane, causing circumlinear striae surrounding the inner edge of the foreign body (Figure 2). There were another two smaller foreign bodies at paracentral 5 and 9 o'clock but away from the visual axis. The seidel test was negative. The anterior chamber was well-formed with minimal reaction seen. There was no hyphaema, pupillary sphincter tear, and lens capsule breach seen. Fundus examination of the right eye showed normal findings. Examination of the anterior and posterior segment of the left eye showed normal findings.

**Figure 3:**
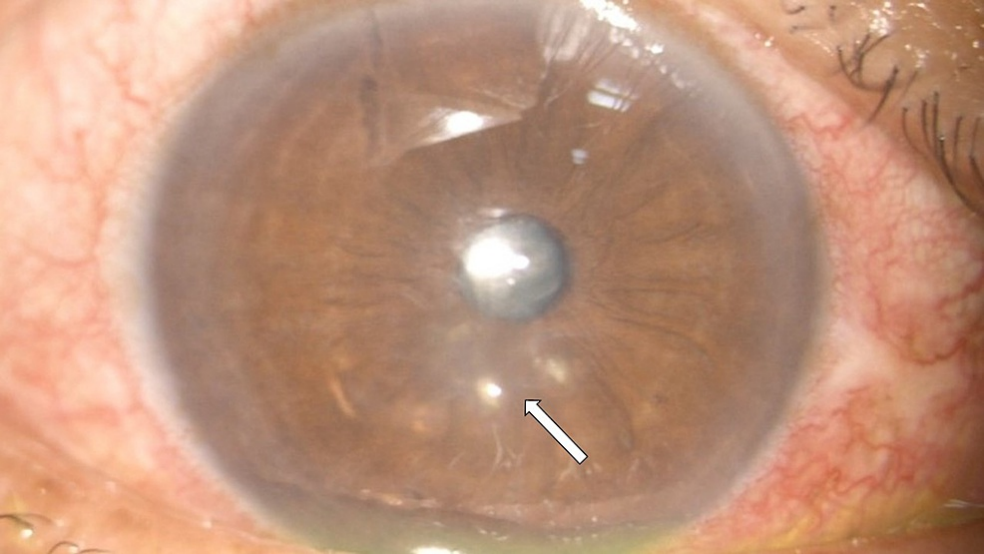
Cornea infiltrates at 5 and 6 clock hours (arrow).

**Figure 4:**
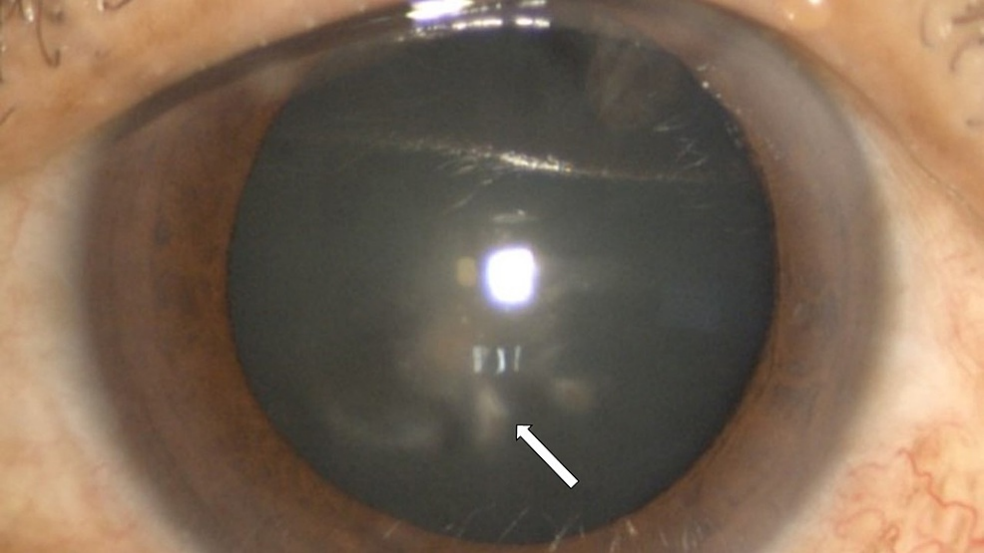
Complete resolution of cornea infiltrates with scarring (arrow).

The patient was given intramuscular anti-tetanus toxoid (ATT) injection prophylaxis. Due to high anticipation of the development of keratitis following cornea trauma by vegetative material, the patient was started on prophylactic topical antibiotic and antifungal which was moxifloxacin 0.5% every four hourly and amphotericin B 0.15% every four hourly respectively. He undergoes right eye corneal foreign bodies removal under general anesthesia. The decision to do the operation under general anesthesia was due to the risk of corneal perforation which might require corneal suturing. Intra-operatively, all the foreign bodies were successfully removed with no evidence of corneal perforation. Post-operatively, the patient was discharged on day 1 and was continued with topical moxifloxacin 0.5% every four hourly and amphotericin B 0.15% every four hourly. During the follow-up on day 4, the patient had developed fungal keratitis over the right eye, evidenced by two areas of small cornea infiltrate at paracentral 6 and 5 o'clock. The infiltrates were less than 0.5 mm in size with feathery edges (Figure 3). However, there was no satellite lesion, endothelial plaques, and hypopyon seen. Posterior segment examination showed an absence of anterior vitreous cell and normal fundus. Although the Gram stain and culture result of the corneal foreign bodies and corneal scrapping were negative for fungal microorganisms, the patient was treated clinically as fungal keratitis due to vegetative-related ocular trauma and the nature of feathery edges of the corneal infiltrate. Subsequently, topical fluconazole 0.2% every four hourly and oral fluconazole 200 mg once daily were added to the existing treatments. Following treatments, the patient showed good response with complete resolution of the cornea infiltrates after two weeks (Figure 4). The visual acuity of the right eye was 6/6 using the Snellen chart. The oral fluconazole was stopped after completing two weeks duration. While the topical medications were slowly tapered in frequency and were stopped after a one-month duration.

## Discussion

Rambutan (Nephelium lappaceum L.) is widely found in warm, humid, and high rainfall regions such as Malaysia, Indonesia, and Thailand [1-2]. The rambutan trees were tall and the fruit grows in a group of sprigs connected by a wooden stalk [1-2]. Due to the height of the tree, it may cause ocular trauma when it accidentally dropped onto the person’s eye, especially when the person is looking up while harvesting. The nature of the ocular trauma can be varied including penetrating ocular injury, sclera cornea laceration wound, ocular foreign body, and blunt trauma. There is no other rambutan-related ocular trauma case reported in the literature, likely due to underreporting.

Previous literature also showed there was the presence of bacteria and fungus colonies in the rambutan [3-4]. Whenever there is a breach in cornea epithelium caused by rambutan-related injury, these microorganisms can invade the eyes and cause serious ocular infections such as keratitis and endophthalmitis.

Generally, treatment of ocular trauma depends on the location and severity of the injury. The location and severity of the ocular injury need to be assessed carefully as it will determine further management [5]. Patients with initial visual acuity which is better than 6/60 were found to have a significantly better visual outcome. On the other hand, patients with more severe ocular injuries such as open globe injuries showed worse visual outcomes [5]. In this patient, as he presented early to the hospital, the removal of deeply embedded corneal foreign bodies was done promptly without corneal perforation. Early surgical intervention is important as prolonged embedded corneal foreign body can cause more damage to the corneal layer and incite a more inflammatory reaction. Early treatment is a critical aspect in managing ocular trauma as it will contribute to visual improvement and minimize ocular complications [5].

The incidence of fungal keratitis is increasingly in trend in developing countries. The incidence is about 17%-44%. Vegetative-related trauma especially in agricultural activities is the most common factor reported in association with fungal keratitis [6-7]. Fusarium species are the most causative organisms, followed by Aspergillus and Candida species [7]. Generally, in determining the visual prognosis of fungal keratitis, patients with better initial visual acuity had better final visual acuity. On the other hand, patients with large infiltrate size and severe fungal keratitis with the presence of hypopyon have more tendency to develop complications such as corneal perforation [6]. Most of the fungal keratitis is treated with combination therapy such as topical amphotericin B and topical fluconazole with oral fluconazole added in severe cases. Other modalities of treatment include intrastromal amphotericin B which is usually prescribed in severe cases who do not respond to initial treatment [6-7]. Early treatment is a crucial stage in the management of fungal keratitis. This is important to prevent the spread of the infection and to reduce the risk of complications. In this patient, due to the nature of ocular trauma involving vegetative material, appropriate broad-spectrum systemic and topical antibiotic and antifungal were started earlier. Even though the patient had developed fungal keratitis following the removal of corneal foreign bodies, the infection was mild and completely resolved following treatment.

## Conclusions

Rambutan fruit may inflict severe ocular trauma and infection. The treating ophthalmologist needs to anticipate the development of fungal keratitis due to the nature of vegetative-related ocular trauma caused by rambutan fruit. Early intervention is crucial to improve visual outcomes and minimize ocular complications. Usage of ocular protection equipment such as goggles is important as it is able to prevent ocular injury especially during harvesting the rambutan fruit.
